# The Alpha-1 Adrenergic Receptor Antagonist Prazosin Reduces Binge-Like Eating in Rats

**DOI:** 10.3390/nu12061569

**Published:** 2020-05-28

**Authors:** Callum Hicks, Valentina Sabino, Pietro Cottone

**Affiliations:** Laboratory of Addictive Disorders, Departments of Pharmacology and Psychiatry, Boston University School of Medicine, Boston, MA 02118, USA; Callum.Hicks@alkermes.com (C.H.); vsabino@bu.edu (V.S.)

**Keywords:** binge-eating disorder, noradrenergic system, addiction

## Abstract

Background: Binge-eating disorder is a pervasive addiction-like disorder that is defined by excessive and uncontrollable consumption of food within brief periods of time. The aim of the current study was to examine the role of the brain noradrenergic system in binge-like eating through the use of the alpha-1 adrenergic receptor antagonist prazosin. Methods: For this purpose, we employed a limited access model whereby male Wistar rats were allowed to nosepoke for either chow (*Chow* rats) or a sugary, highly palatable food (*Palatable* rats) for 1 h/day. The effects of prazosin (0, 0.5, 1 and 2 mg/kg, i.p.) were tested in a fixed ratio 1 (FR1) and progressive ratio (PR) schedule of reinforcement. Results: The results show that prazosin preferentially reduced the responses for palatable food in a FR1 reinforcement schedule; when tested in a PR schedule of reinforcement, prazosin increased breakpoint in both *Chow* and *Palatable* rats, but more potently and more efficaciously in the latter. Our results suggest that prazosin treatment preferentially increased the motivational properties of the palatable diet. Conclusions: The current findings provide the characterization of the effects of prazosin on binge-like eating and offer support to the existing literature showing the important role of the noradrenergic system in addiction-like behavior.

## 1. Introduction

Binge-eating disorder (BED) currently affects approximately 8 million people in the United States, and is characterized by excessive and uncontrollable consumption of food within brief periods of time (Diagnostic and Statistical Manual of Mental Disorders, 5th ed. (DSM–5); [1]. Accumulating evidence indicates that BED displays the hallmarks of an addiction-like disorder, as many of the core symptoms of binge eating mirror those seen in drug-addicted individuals [2]. Moreover, evidence suggests that binge eating is associated with neuroadaptations in discrete brain regions that are also present in drug and alcohol addiction [3].

The brain noradrenergic system is well known to play a central role in drug and alcohol abuse and has been implicated in all stages of drug addiction [4]. Interestingly, the noradrenergic system has also been linked to the excessive consumption of highly palatable foods in eating disorders [5]. Indeed, this system stimulates the preferential intake of sugar- and/or fat-rich highly palatable foods, and conversely consumption of palatable foods increases central release of noradrenaline [6].

Prazosin is a centrally acting alpha-1 adrenergic receptor antagonist that has been shown to modulate addiction-like behaviors. Prazosin has been shown to reduce alcohol consumption in both a genetic [7] and environmental [8] model of alcoholism, to inhibit drug-primed reinstatement of cocaine-seeking behavior [9] and to reduce heroin self-administration in rats given extended access to the drug [10]. Importantly, however, to the best of our knowledge, the effects of prazosin specifically on binge eating have not yet been examined.

In the current study, we therefore aimed to determine the effects of systemically administered prazosin on binge-like eating in male rats induced by limiting access to highly palatable food [11,12,13,14,15,16,17]. We used a self-administration model of binge-like eating and employed a fixed ratio 1 (FR1) and progressive ratio (PR) schedule of reinforcement.

## 2. Materials and Methods

### 2.1. Animals

Male Wistar rats obtained from Charles River Laboratories (Wilmington, MA) were aged 45 days upon arrival and double- or triple-housed in a humidity- (60 ± 2%) and temperature-controlled (22 ± 1 °C) vivarium maintained on a reverse 12:12-h light/dark cycle (lights off at 11:00-h). Upon arrival, rats were given *ad libitum* access to water and standard laboratory chow. After a period of acclimation, the standard chow was replaced with an AIN-76A-based diet (5TUM diet formulated as 4–5 g extruded pellets; TestDiet, Richmond, IN), hereafter referred to as ‘Chow A/I’. All procedures adhered to the NIH Guide for the Care and Use of Laboratory Animals and the Principles of Laboratory Animal Care, and were approved by the Boston University Medical Campus Institutional Animal Care and Use Committee.

### 2.2. Drugs

Prazosin hydrochloride was purchased from Alfa Aesar (Ward Hill, MA) and was dissolved in a 10% dimethyl sulfoxide (DMSO) and 90% filtered sterile water vehicle. It was given by intraperitoneal (i.p.) injection at a volume of 1 mL/kg. The doses of prazosin to be administered (0.5, 1 and 2 mg/kg) were based on previous studies [8,10,18].

### 2.3. Operant Binge-Like Eating Procedure

The rats (*n* = 39) were allowed to self-administer food and water (100 μL) in daily 1-h sessions in individual operant test chambers previously described in detail by Cottone et al. [19]. All sessions were conducted prior to the onset of the dark cycle. For the FR1 and PR tests, prazosin was administered using a within-subject Latin-square design 30 min prior to the start of the binge sessions. Drug injections were separated by 1–3 washout days after ensuring that responding had returned to baseline.

### 2.4. Training Phase

The rats were trained on a FR1 reinforcement schedule to nosepoke for 45 mg precision food pellets (5TUM diet; TestDiet, Richmond, IN, USA) that were identical to the home cage ~5 g extruded diet. This was to ensure that the food intake of *Chow* rats was driven exclusively by homeostatic needs [19,20].

### 2.5. Fixed Ratio 1 Testing

Once responding in the training phase had stabilized, rats were separated into two groups, ‘*Chow*’ (*n* = 19) and ‘*Palatable*’ (*n* = 20), matched for body weight, food and water intake in the self-administration sessions. *Chow* rats served as the control group and received the same 45 mg chow pellets used in the training phase. The *Palatable* group, on the other hand, were given a chocolate-flavored high sucrose (50% kcal) AIN-76A-based diet (5TUL diet formulated as 45 mg precision food pellets; TestDiet, Richmond, IN), comparable in macronutrient composition and energy density to the chow diet. We have shown that rats strongly prefer this chocolate-flavored diet [19,21]. Drug testing commenced once responding in both groups had stabilized.

### 2.6. Progressive Ratio Testing

Once testing under FR1 was complete, rats were trained on a PR schedule of reinforcement. The PR sessions employed the same general procedure and length (1 hour) as the FR1 sessions, except that the number of responses required to produce a food pellet increased with successive food reinforcers based on the following shallow exponential progression: response ratio = [4·(e^# of reinforcer*0.075^) − 3.8], rounded to the nearest integer for more details, see [16,19]. Drug injections commenced after the response rates of *Chow* (*n* = 19) and *Palatable* (*n* = 20) rats had stabilized.

### 2.7. Locomotor Activity Test

To determine whether there were any non-specific behavioral disturbances on food responses as a result of prazosin treatment, rats were tested in a 1-h locomotor activity test as previously described [15]. The rats were tested for locomotor activity on two occasions, with vehicle and 2 mg/kg prazosin administered in a within-subject, Latin-square design. The drugs were injected 30 min prior to the start of the test, and the injections were separated by 2 washout days to ensure adequate drug clearance.

### 2.8. Statistical Analysis

The effects of prazosin on FR1 water responses were analyzed using a two-way mixed design analysis of variance (ANOVA). FR1 food responses, PR breakpoint, and locomotor counts were measured across six 10 min bins and the incremental values were analyzed using a three-way mixed design ANOVA. The cumulative values for each group were analyzed by one-way repeated measures ANOVAs with Bonferroni post-hoc analyses. Homogeneity of variance was assessed and when this requirement was not satisfied, the variables were analyzed as ranked values. All analyses were performed using SPSS v. 19 (SPSS Inc., IBM, Chicago, IL) with significance set at *p* < 0.05.

## 3. Results

### 3.1. Prazosin Effects on FR1 Responding

**Figure 1:**
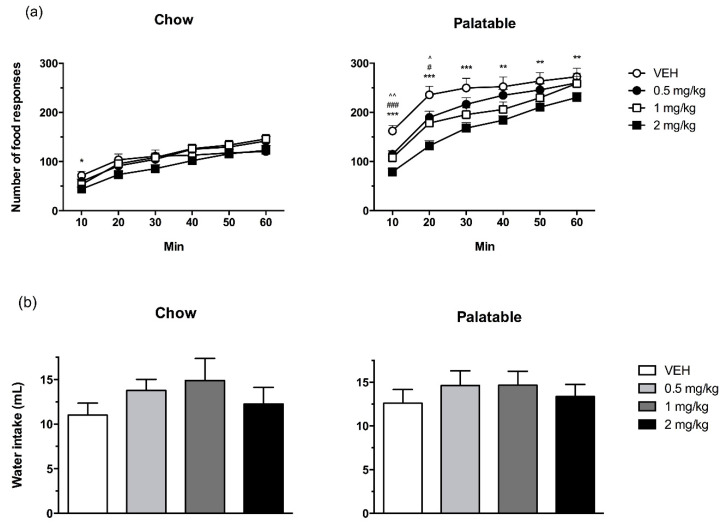
The effects of prazosin on food responses’ time course (**a**) and cumulative water intake (**b**) in *Chow* and *Palatable* rats in a fixed ratio 1 reinforcement schedule. Prazosin produced a more pronounced reduction in food responses in *Palatable* relative to *Chow* rats, but did not significantly affect water intake in either group. Data are presented as mean + SEM. Symbols indicate a significant difference from vehicle (VEH) within each group; 0.5 mg/kg: ^ *p* < 0.05, ^^ *p* < 0.01; 1 mg/kg: ^#^ *p* < 0.05, ^###^ *p* ≤ 0.001; 2 mg/kg: * *p* < 0.05, ** *p* < 0.01, *** *p* < 0.001.

Although *Palatable* rats, overall, made a greater number of food responses compared to *Chow* rats (Figure 1a; diet history: *F*(1,37) = 11.53, *p* < 0.01), prazosin reduced responses irrespective of diet history (treatment: *F*(3,111) = 16.78, *p* < 0.001) (diet history × treatment: *F*(3,111) = 0.20, (*n.s.*). The analysis of the time-course revealed that the effects of prazosin were time-dependent (treatment x time: *F*(15,555) = 3.64, *p* < 0.001). Post-hoc tests showed that 2 mg/kg prazosin significantly reduced the food responses of *Chow* rats in the first 10 min, compared to vehicle. In *Palatable* rats, prazosin significantly affected the number of food responses at all time points examined; in the first 10 and 20 min, all 3 doses of prazosin reduced responses relative to vehicle treatment. In addition, the 2 mg/kg dose continued to reduce responding for the remainder of the session.

*Chow* and *Palatable* rats did not differ in their responses for water, and prazosin treatment did not reliably affect this measure (Figure 1b; diet history: *F*(1,37) = 1.01, *n.s.*; treatment: *F*(3,111) = 2.64, *n.s.*; diet history x treatment: *F*(3,111) = 0.29, (*n.s.*)).

### 3.2. Prazosin Effects on PR Responding

**Figure 2:**
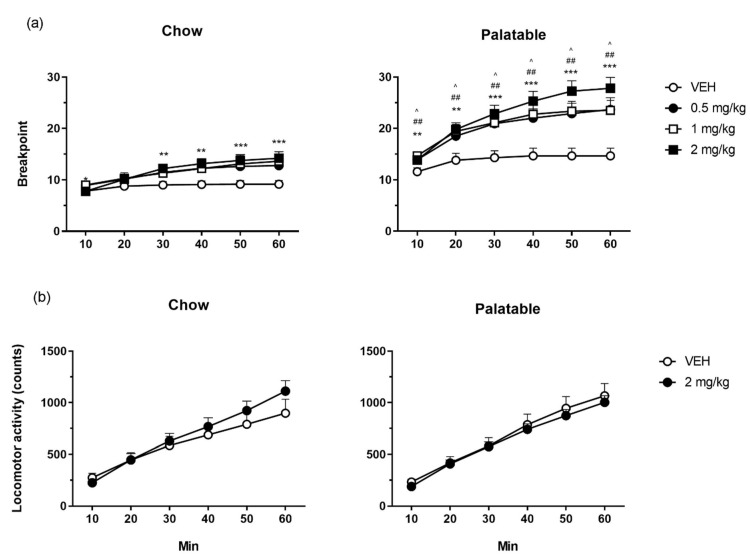
The effects of prazosin on breakpoint time course in a progressive ratio schedule of reinforcement task (**a**) and on counts’ time course in a locomotor activity test (**b**), in *Chow* and *Palatable* rats. Prazosin dose-dependently increased PR breakpoint, more potently and more efficaciously in *Palatable* rats than *Chow* rats, without significantly altering locomotor activity levels. The data are presented as mean + SEM. Symbols indicate a significant difference from vehicle (VEH) within each group; 0.5 mg/kg: ^ *p* < 0.05; 1 mg/kg: ^##^ *p* ≤ 0.01; 2 mg/kg: ** *p* < 0.01, *** *p* < 0.001.

Overall, the breakpoint of the *Palatable* rats was higher than that of *Chow* rats (Figure 2a; diet history: *F*(1,37) = 27.94, *p* < 0.001). Moreover, breakpoint was increased by prazosin preferentially in *Palatable* rats as compared to *Chow* rats (treatment: *F*(3,111) = 18.23, *p* < 0.001; diet history x treatment: *F*(3,111) = 3.43, *p* < 0.02). The analysis of the time-course revealed that the effects of prazosin were time-dependent (treatment × time: *F*(15,555) = 2.61, *p* < 0.001). Post-hoc tests revealed that prazosin increased breakpoint in *Palatable* rats at all doses tested since the first 10 minutes of test and lasted throughout the entire session. On the other hand, prazosin increased the breakpoint in *Chow* rats only at the highest dose tested (2 mg/kg) starting from the 30-minute bin and until the end of the session.

### 3.3. Prazosin Effects on Locomotor Activity

*Chow* and *Palatable* rats did not differ significantly in locomotor activity (Figure 2b; diet history: *F*(1,35) = 0.07, *n.s.*). There was also no significant effect of prazosin on activity irrespective of diet history and time (treatment: *F*(1,35) = 0.55, *n.s.*; diet history x treatment: *F*(1,35) = 1.91, *n.s.*; time (diet history x treatment: *F*(5,175) = 1.39, *n.s.*).

## 4. Discussion

We show that systemic administration of the alpha-1 adrenergic receptor antagonist prazosin modulates the motivational component of feeding in an operant model of binge eating. Specifically, prazosin produced a more marked reduction in the number of food responses of *Palatable* rats as compared to *Chow* rats under an FR1 reinforcement schedule, while at the same time increased the motivation of *Palatable* rats to work to obtain food on a PR schedule more potently and more efficaciously than *Chow* rats. These effects occurred independently of any changes in general behavioral activation as prazosin did not significantly affect water intake, nor did it alter locomotor activity, consistent with what has been shown previously [22].

A time bin analysis of FR1 food responding revealed that prazosin reduced palatable food intake at all doses tested. Drug treatment affected FR1 chow responding as well, but very transiently during the first 10 min of the session and only at the highest dose administered (i.e., 2 mg/kg).

Prazosin increased motivation for food irrespective of the type of diet self-administered; however, drug treatment was more potent and more efficacious in *Palatable* rats as compared to *Chow* rats. Indeed, prazosin increased the breakpoint in *Palatable* rats at all doses tested, since the first 10 min bin, and up to 89.8% of the vehicle condition, while the breakpoint in *Chow* rats increased only following administration of the highest dose in the second half of the session, and only up to 55.2% of the vehicle condition. Despite the relatively abundant literature on the effects of prazosin on self-administration behavior on an FR1 schedule, the reports on the effects on PR are sparser and to the best of our knowledge, the effects of prazosin in a PR schedule of reinforcement for food have, to our knowledge, not yet been reported.

The opposite effects of prazosin on FR1 and PR responding may, at first, seem conflicting. However, several drug manipulations have been shown to have an effect on PR which is inversely related to their effect on FR1, such that drugs that increase reinforcing efficacy of a substance reduce their self-administration in FR1, and vice versa [23,24,25,26,27,28,29,30,31,32,33,34]. In such cases, while changes in self-administration behavior on an FR1 schedule can be difficult to interpret, alterations in reinforcing efficacy can be better understood using PR schedules [27,28]. In our experiments we show that prazosin increases overall the breakpoint for food, with greater potency and efficacy that for the palatable diet as compared to chow. This effect is by itself a direct evidence for an increase of reinforcing efficacy of food induced by prazosin treatment. Therefore, in absence of any other locomotor, time-related, or rebound effect, the most plausible interpretation of the decrease in high-palatable food self-administration observed in FR1 following prazosin treatment is a compensatory response to an increase in the reinforcing efficacy of food (as measured by breakpoint in PR) [27,28]. A potential argument against this interpretation is that reinforcing efficacy in PR should be affected only by substances which have per se rewarding/reinforcing effects [23,24,25,26], while prazosin does not appear to [35]. The counterargument is that not all drugs that increase a substance’s reinforcing efficacy are rewarding/reinforcing per se [30,36,37,38,39].

The current findings add to an increasing body of literature showing a central role for the brain noradrenergic system in the excessive consumption of highly palatable foods. Prazosin has previously been reported to attenuate cocaine-induced hypophagia [22], reduce self-administration of sucrose solutions [40,41] and inhibit stress-induced reinstatement of food seeking [18]. Moreover, previous studies show that drugs that act as agonists at the alpha-1 adrenergic receptor [42], or block the reuptake of noradrenaline [43,44], reliably induce hypophagia.

A limitation of this study is related to the fact that only male subjects were used. Future studies will be needed to investigate any sex differences in the effects of prazosin through direct comparison between female and male subjects.

In summary, using a limited access model we show that systemic administration of the alpha-1 adrenergic receptor antagonist prazosin dose-dependently decreases palatable food self-administration in FR1 in a binge-like eating model and increased reinforcing efficacy of palatable food, as measured by PR. Prazosin yielded more pronounced effects on the maladaptive feeding behavior of male rats given highly palatable food in comparison to those given regular chow. The present findings provide further evidence of the involvement of the brain noradrenergic system in binge eating and contribute to an expanding body of literature showing a central role for this system in addictive behaviors.
